# Burden of invasive pneumococcal disease among children in rural Mozambique: 2001-2012

**DOI:** 10.1371/journal.pone.0190687

**Published:** 2018-01-05

**Authors:** Betuel Sigaúque, Jennifer R. Verani, Sérgio Massora, Delfino Vubil, Llorenç Quintó, Sozinho Acácio, Inácio Mandomando, Quique Bassat, Tacilta Nhampossa, Fabiana Pimenta, Charfudin Sacoor, Maria da Gloria Carvalho, Eusebio Macete, Pedro L. Alonso

**Affiliations:** 1 Fundação Manhiça / Centro de Investigação em Saúde da Manhiça (CISM); Maputo, Mozambique; 2 Instituto Nacional de Saúde, Ministério de Saúde, Maputo, Mozambique; 3 Respiratory Diseases Branch, Centers for Disease Control and Prevention, Atlanta, Georgia, United States of America; 4 ISGlobal, Barcelona Center of International Health Research (CRESIB), Hospital Clínic—Universitat de Barcelona, Barcelona, Spain; Universidade de Lisboa Faculdade de Medicina, PORTUGAL

## Abstract

**Background:**

Invasive pneumococcal disease (IPD) is a major cause of illness and death among children worldwide. 10-valent pneumococcal conjugate vaccine (PCV10) was introduced as part of the Mozambican routine immunization program in April 2013. We characterized the IPD burden in a rural area of Mozambique before PCV introduction and estimated the potential impact of this intervention.

**Methods:**

We conducted population-based surveillance for IPD, defined as *S*. *pneumoniae* isolated from blood or cerebrospinal fluid, among children <5 years old admitted to Manhiça District Hospital, a referral hospital in a rural area with high prevalence of human immunodiciency virus infection. *S*. *pneumoniae* was identified using standard microbiologic methods and serotyped using sequential multiplex PCR or Quellung. IPD incidence was calculated among cases from a defined catchment area.

**Results:**

From January 2001 through December 2012, we isolated 768 cases of IPD, 498 (65%) of which were bacteraemic pneumonia episodes. A total of 391 (51%) were from the catchment area, yielding IPD incidence rates of 479, 390 and 107 episodes per 100,000 children-years at risk among children <12, 12–23 and 24-<60 months old, respectively. The overall IPD incidence fluctuated and showed a downward trend over time. In these same age groups, in-hospital death occurred in 48 (17%), 26 (12%), and 21 (13%) of all IPD cases, respectively. Overall 90% (543/603) of IPD isolates were available for serotyping; of those, 65% were covered by PCV10 and 83% by PCV13. Among 77 hospital deaths associated with serotyped IPD, 49% and 69% were caused by isolates included in the PCV10 and PCV13, respectively.

**Conclusions:**

We describe very high rates of IPD among children in rural Mozambique that were declining before PCV introduction. Children <1 year old have the greatest incidence and case fatality; although the rates remain high among older groups as well. Most IPD episodes and many deaths among children <5 years old will likely be prevented through PCV10 introduction in Mozambique.

## Introduction

*Streptococcus pneumoniae* is a leading cause of bacterial pneumonia, bacteremia/sepsis, and acute meningitis in children worldwide [1]. The World Health Organization (WHO) estimated that approximately 541,000 children less than 5 years old died in 2008 of pneumococcal disease [2], and most pediatric pneumococcal deaths occur in developing countries [1]. In 2007, based on demonstrated efficacy and safety of a 7-valent and a 9-valent pneumococcal conjugate vaccine (PCV) [3–6], WHO recommended that PCV be included in routine immunization programs, especially in countries with high mortality rates among children under the age of 5 years and high prevalence of HIV infection [7]. In 2009 and 2010, 10-valent (PCV10) and 13-valent (PCV13) PCVs were licensed, offering improved coverage for prevalent pneumococcal serotypes, particularly in Africa where serotypes 1 and 5 are frequent [8]. PCVs have been increasingly introduced in sub-Saharan Africa in recent years [9]. However, due to difficulties in building the necessary laboratory and epidemiologic capacities, few African settings have high-quality population-based surveillance for invasive pneumococcal disease.

Since 2001, the *Centro de Investigação em Saúde de Manhiça* (CISM) has been conducting surveillance for invasive bacterial disease among children in rural Mozambique [10–12]. Data from this surveillance system were instrumental in prompting the Mozambican Ministry of Health to apply for GAVI Alliance support to introduce Hib conjugate vaccine in August, 2009 and PCV10 in April, 2013. PCV10 was introduced using a 3-dose schedule (given at 2, 3, and 4 months of age) with no catch-up campaign. We examined pre-PCV surveillance data in order to describe the burden of invasive pneumococcal disease (IPD) before vaccine introduction and to estimate the anticipated impact of PCV introduction on pneumococcal illness and death among children in Mozambique.

## Material and methods

### Study area and population

Surveillance for invasive pneumococcal disease (IPD) is routinely conducted at Manhiça District Hospital (MDH), a referral district hospital in Southern Mozambique. Details of the study site have been described elsewhere [13]. Manhiça district has a total population of approximately 165,000 persons, 18% of which are children <5 years old [14]. The area is rural, malaria-endemic [15] and with a high burden of human immunodeficiency virus (HIV); HIV prevalence among pregnant woman was estimated to be 24% in 2003 to 2005 [16] and overall community prevalence peaked at 39% in 2011 [17]. Within the district, CISM has maintained a continuous demographic surveillance system (DSS) since 1996, recording life events (pregnancy, births, deaths and migration in or out of the study area) and performing updates biannually from 1996 to 2012. The DSS area initially included approximately 36,000 people; by 2005 it had been expanded to include 92,000 people; which correspond to 58% of the total district population in Manhiça [13].

The protocol for population-based invasive pneumococcal surveillance described in this study was reviewed and approved by the Mozambican National Bioethics Committee, the Institutional Review Board of the Clinic Hospital of Barcelona (Barcelona, Spain), and was determined to be non-human subjects research by the U.S. Centers for Disease Control and Prevention.

### Morbidity surveillance and sample collection

Since January 1997, CISM has operated pediatric morbidity surveillance at MDH and within a network of 5 peripheral health centers within the DSS area [10, 12]. In these health facilities, trained clinical personnel perform physical examinations and complete standardized forms to record demographic information, presenting signs and symptoms, and clinical outcomes. During the study period, HIV testing results were not routinely available; because of concern about stigma. HIV testing was often performed anonymously and therefore the results are not linkable to the IPD surveillance database. At MDH, the only site with admission facilities and linked to demographic surveillance, 1–3 mL of blood for culture are obtained by venipuncture upon admission and before administration of antimicrobials for all children <2 years requiring hospitalization and for those 2-<15 years with axillary temperature ≥39°C or other signs of severe disease. Lumbar puncture to collect cerebrospinal fluid (CSF) is systematically performed on all children with suspected meningitis and admitted infants <29 days old.

### Bacteriological procedures

Pediatric blood culture bottles (Pedibact®, Becton-Dickinson, Franklin Lakes, NJ) were inoculated with 1–3 ml of whole blood and incubated in an automated system (BACTEC® 9050 Becton-Dickinson, Franklin Lakes, NJ) for 4 days. Post-incubation specimens from blood culture bottles with a Gram stain compatible with pneumococcus were sub-cultured onto blood agar and incubated overnight at 37°C in 5% CO_2_. CSF samples were first stained by Gram and subsequently cultured onto blood agar, chocolate blood agar and thioglycolate broth media for 72h, at 37°C in an atmosphere of 5% CO2. Thioglycolate broths were subcultured on chocolate blood agar media and incubated for 72h. Pneumococci were identified by α-hemolysis and typical colony morphology and confirmed by optochin sensitivity.

Serotyping of pneumococcal isolates was consistently performed starting in 2003. Pneumococcal strains were initially serotyped at the CDC *Streptococcus* laboratory by Quellung reaction with serotype-specific antiserum [18]. Since 2006, serotyping has been performed at CISM using sequential multiplex PCR [19], with quality control and resolution of serogroups that cannot be differentiated by PCR done at CDC in Atlanta by Quellung method.

All pneumococcal isolates from 2008 to 2010 were tested for antibiotic susceptibility using broth microdilution method. Isolates were classified as susceptible, intermediate or resistant according to the definitions of the National Committee for Clinical and Laboratory Standards Institute (CLSI) for bacteremia [20] for 2011.

### Definitions

A case of IPD was defined as the isolation of *S*. *pneumoniae* from a normally sterile site (such as blood or CSF) in a child <5 years of age presenting to MDH. Cases with *S*. *pneumoniae* detected in CSF (CSF only or in both CSF and blood) were classified as pneumococcal meningitis; all other cases with *S*. *pneumoniae* in the blood were classified as bacteraemia. Among bacteraemia cases, those with cough and/or breathing difficulties plus tachypnea were classified as bacteraemic pneumonia. Case-fatality for IPD episodes excludes patients with unknown outcome; 90 children out of 670 (because were referred to referral hospital or absconded from the hospital), and thus represents in-hospital mortality. PCV10 serotypes include 1, 4, 5, 6B, 7F, 9V, 14, 18C, 19F and 23F; because of potential cross-protection against serotype 6A [21], we also examined PCV10 serotypes plus 6A. PCV13 includes the same pneumococcal serotypes included in the PCV10 plus 3, 6A and 19A.Weight-for-age z-score was calculated using WHO child growth chart standards [22].

### Data management and analysis

Data from standardized forms were double entered using visual Fox Pro version 2.6 (Microsoft Corporation, Redmond, WA) at CISM. Proportions were compared using the *X*^2^ test. We calculated the incidence rates of IPD, pneumococcal bacteremia and pneumococcal meningitis among children <1 year, 1–2 years, 2-<5 years from the DSS area. Recurrent detection of *S*. *pneumoniae* within 14 days of the first positive culture was considered the same episode of IPD. To estimate incidence, person-time of follow-up for children in the demographic surveillance area was calculated per 100,000 children-years at risk (CYR) using dates of birth and death, excluding periods of outmigration. Negative binomial regression models were developed to estimate incidence rates overall and by age-group. Models were estimated with random intercept to take into account repeated measures, since children can belong to several age categories or to several calendar years during the surveillance period.

Statistical analyses were performed using STATA software (version 12.0, STATA Corporation, College Station, TX).

## Results

From 1^st^ January 2001 to 31^st^ December 2012, there were 41,106 admissions to MDH among children <5 years of age, of which 48% were from the DSS catchment area. Per hospital protocol, blood culture was indicated for nearly all admitted children (n = 41,032, 99.8%); and it was performed for 34,947 (85%) of admissions with an indication. CSF culture was done for 3,347 patients, representing 8% of all admissions and 56% (3,347/6,006) of those with an indication for lumbar puncture per hospital protocol. The proportion of eligible patients undergoing lumbar puncture was lowest during the first two years of surveillance (569/1,264, 45%); in more recent years the proportion ranged from 82% to 85%.

A total of 768 IPD cases were identified, including 498 (65%) bacteraemic pneumonia episodes, 218 (28%) bacteraemia (non-pneumonia) cases and 52 (7%) pneumococcal meningitis cases (Table 1). Among pneumococcal meningitis cases, 35 (67%) had *S*. *pneumoniae* isolated from blood in addition to being detected in the CSF. Overall, 55% (425/768) of IPD cases were in males and 76% (584/768) occurred among children under the age of <24 months. Among 64 cases (8%) with documented HIV status, 34 (53%) occurred in HIV-infected children. Among 667 cases with known nutritional status, 159 (24%) had severe malnutrition. Hospital death occurred in 48 (17%), 26 (12%), and 21 (13%) of all cases aged <12 months, 12–23 months and 24-<60 months old respectively (p = 0.078). Overall case fatality was 14% (95/670), and significantly higher among pneumococcal meningitis cases (38%; 15/40) compared with non-meningitis syndromes (13%, 80/630; p = 0.001), among cases with known outcomes. Almost half (51%; 48/95) of IPD death occurred among infants.

**Table 1:** Characteristics of IPD cases (n = 768) detected from January 2001 to December 2012 at Manhiça District Hospital, in Mozambique.

Characteristics	Bacteraemic pneumonia	Bacteraemia (non-Pneumonia)	Meningitis	Overall
	n = 498	n = 218	n = 52	N = 768
	n (%)	n (%)	n (%)	n (%)
**Gender:** male	269 (54)	127 (58)	29 (56)	425 (55)
**Age (months)**				
Median (interquartile range)	14 (8 to 24)	11 (7 to 20)	8.5 (4 to 18)	13 (7 to 23)
0–11	195 (39)	111 (51)	30 (58)	336 (44)
12–23	174 (35)	62 (28)	12 (23)	248 (32)
24-<60	129 (26)	45 (21)	10 (19)	184 (24)
Malnutrition*	135 (30)	48 (27)	22 (45)	22 (45)
None	135 (30)	48 (27)	22 (45)	22 (45)
Mild	86 (20)	51 (28)	13 (27)	150 (23)
Moderate	104 (24)	38 (21)	11 (22)	153 (23)
Severe	112 (26)	44 (24)	3 (6)	159 (24)
**Seasonality**				
Rainy season (October–March)	234 (47)	96 (44)	26 (50)	356 (46)
**Hospitalized duration (days)**				
Median (interquartile range)	6 (3 to 9)	5 (3 to 8)	3 (2 to 5)	5 (3 to 9)
Case fatality by age group**				
0–11 months	24/169 (14)	12/97(12)	12/24(50)	48/290 (17)
12–23 months	18/151 (12)	6/52 (12)	2/9 (22)	26/212 (12)
24-<60 months	17/119 (14)	3/42 (7)	1/7 (14)	21/168 (13)
Overall	59/439 (14)	21/191 (11)	15/40 (38)	95/670 (14)

*Among for 667 IPD cases with complete weight or age data for weight for age Z-score (waz) calculation (waz >-2 to <-1 Mild; waz>-3 to <-2 Moderate; waz<-3 severe).

** Among 670 IPD cases with known outcome (missing date in 47 among infants (<12 months), 36 among children age 12-<24m and 16 among children age 24-<60).

**Fig 1:**
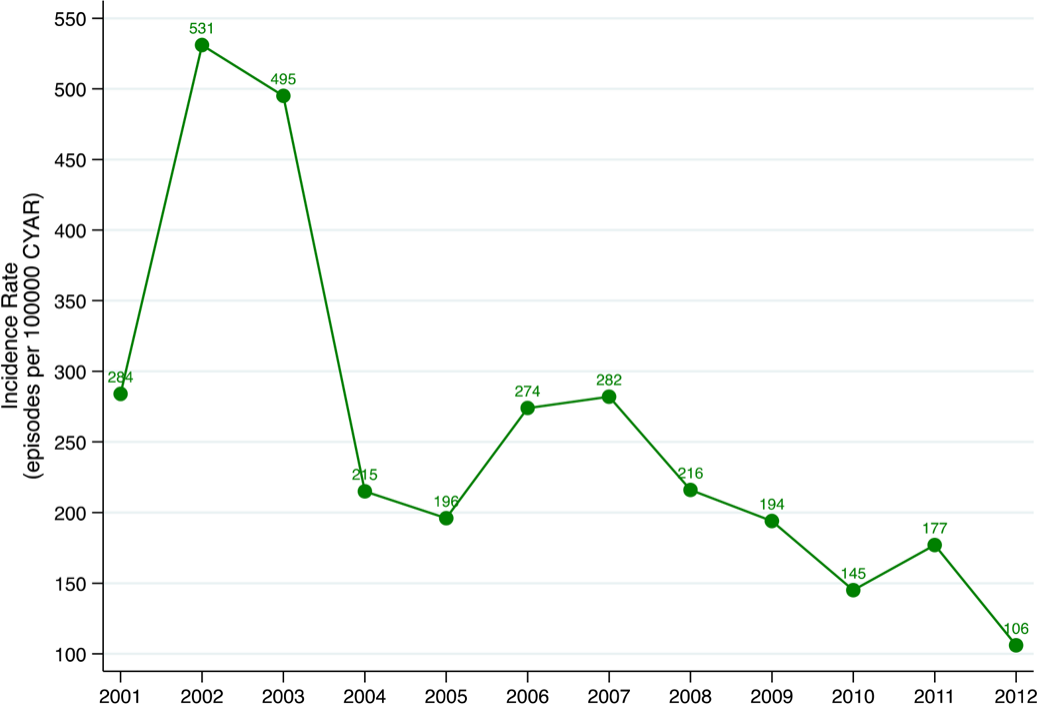
Annual incidence rates of IPD cases among children <5 years of age admitted at Manhiça District Hospital, in Mozambique between 2001–2012.

**Incidence rate**: Among 391 IPD cases from the DSS area, 4 were in children who had a positive pneumococcal culture within the prior 15 days; thus a total of 387 unique IPD episodes were detected. The overall incidence was 245 episodes per 100,000 CYR (Table 2). Rates were highest among infants 0–11 months (479 episodes per 100,000 CYR) and among children aged 12–23 months (390 episodes per 100,000 CYR). Incidence rates for all children <5 years varied over time, with a peak incidence of 531 episodes per 100,000 CYR in 2002–2003 (Fig 1). Overall, during the study period there was a trend toward declining incidence rates, with the lowest rate observed in 2012 (106 episodes per 100,000 CYR).

**Table 2:** Incidence rate of IPD among children <5 years old in rural Mozambique per different age groups, in Manhiça District Hospital.

Age groups (months)	Episodes	Time at risk	Incidence	IRR^#^	95%
		(CYR)	(Episodes per 100,000 CYR*)		Conf. Interval
0–11	163	34,018	**479**	1	
12–23	126	32,345	**390**	0.82	(0.65–1.04)
24–59	98	91,848	**107**	0.23	(0.17–0.29)
**TOTAL**	**387**	**158,211**	**245**	-	-

* CAR = children-years at risk.

**#** IRR = Incidence Rate Ratio.

**Fig 2:**
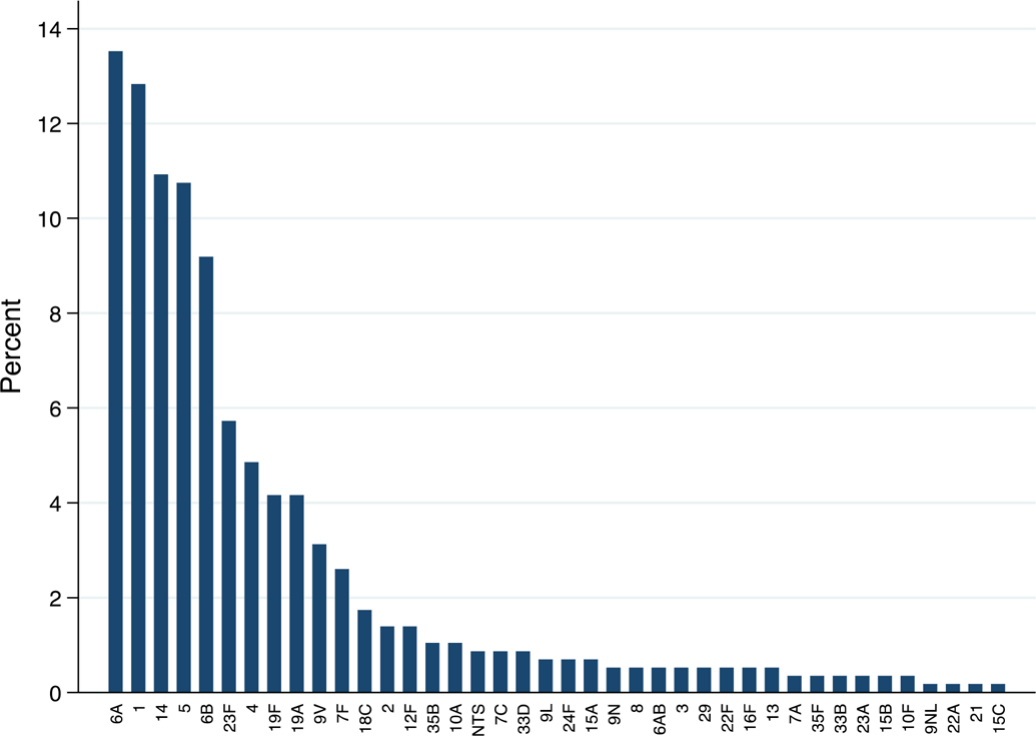
Serotypes distribution of pneumococcal isolates (n = 546) in blood and CSF of children <5 years of age admitted at Manhiça District Hospital, in Mozambique: 2003–2012.

**Fig 3:**
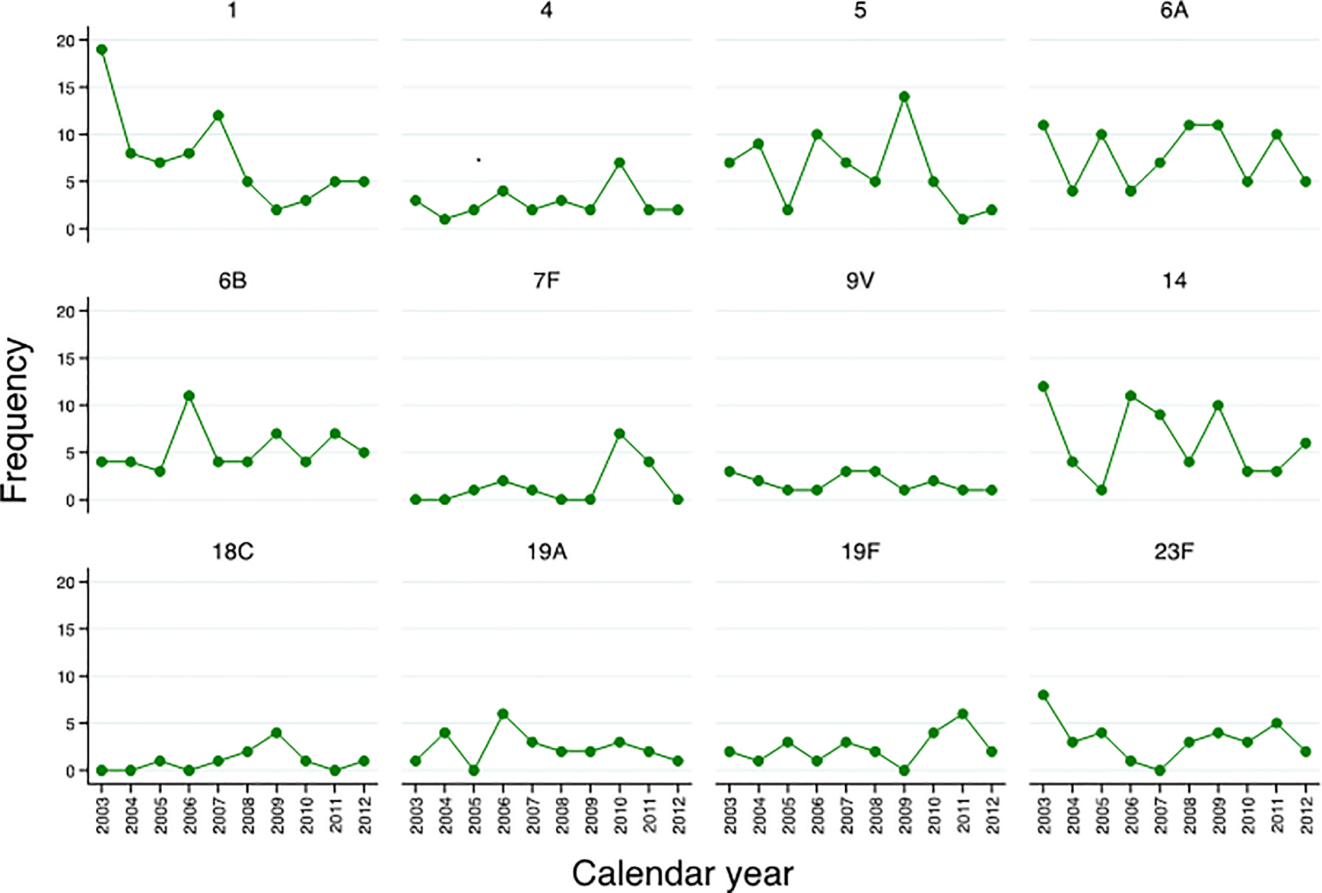
Number of IPD cases caused by most common vaccine types among children <5 years of age admitted at Manhica District Hospital, 2003–2012.

**Serotypes**: From 603 IPD episodes occurring in 2003–2012, 543 (90%) isolates were available for serotyping. Among those, 5 (1%) were non-typable. Among the remaining 538, 37 different serotypes were detected. The most prevalent serotypes were 6A (13.9%), 1 (13.2%), 14 (11.2%), 5 (10.7%), 6B (9.0%), and 23F (6.1%), (Fig 2). Three (<1%) isolates were classified as 6AB by PCR but no isolate was available to definitively determine serotype by Quellung. The relative contribution of different serotypes varied over time, with the greatest number of serotypes 1, 14 and 23F cases observed in 2003 and in 2009 the greatest number it was recorded for serotypes 5, 6A and 14 (Fig 3).

The proportion of cases due to serotypes included in PCV10 and PCV13 were 65% and 83%, respectively, with the difference attributable primarily to serotypes 6A and 19A. If we assume cross protection against 6A from PCV10, then the proportion of cases covered by PCV10 increases to 78%. Serotype coverage did not vary significantly by clinical syndrome, age group or by year (Table 3). Serotype data were available for 77 IPD in-hospital deaths; 38 (49%) were due to serotypes included in PCV10 and 52 (69%) due to serotypes included in PCV13.

**Table 3:** Proportion of pneumococcal disease syndromes potentially preventable by PCV formulation, among children < 5 years of age in Mozambique.

	PCV10		PCV13	
	n/N	%	n/N	%
Overall	383/588	65	488/588	83
Clinical syndromes				
Bacteraemic pneumonia	247/375	66	310/375	83
Bacteraemia (non-pneumonia)	105/165	64	141/165	85
Pneumococcal Meningitis	31/48	65	37/48	77
Age group				
0–11 months	166/263	63	210/263	80
12–23 months	118/187	63	159/187	85
24–59 months	99/138	72	119/138	86

**Antimicrobial susceptibility:** Table 4 summarizes pneumococcal antimicrobial resistance of 195 IPD isolates between 2008 and 2010. Resistance to cotrimoxazole was most common (54.4%), followed by tetracycline (23.6%) and chloramphenicol (14.4%). Resistance to beta lactam antibiotics was low, with <1% resistant to penicillin or ampicillin. Among 106 isolates resistant to cotrimoxizale, 68 (64%) were vaccine serotypes.

**Table 4:** Antibmicrobial susceptibility for 195 invasive pneumococcal isolates with antimicrobila susecptability performed from children <5 years old, hospitalized at Manhiça District Hospital, Mozambique.

Antibiotics	Intermediate*	Resistant*	Range MICs
	*n* (%)	*n* (%)	
Erythromycin	0	7 (3.6)	≤0.03 to >32
Penicillin #	0	1 (0.5)	≤0.03 to 4
Amoxicillin	0	1 (0.5)	≤0.03 to 4
Cefotaxime	0	0	≤0.06 to 1
Clindamycin	0	3 (1.5)	≤0.03 to >2
Cotrimoxazole	57 (29.2)	106 (54.4)	≤0.12 to >4
Chloramphenicol	-	28 (14.4)	≤2 to >8
Tetracycline	0	46 (23.6)	≤2 to >8

# Using CLSI 2011 breakpoints 4ug/ml for intermediate and ≥8ug/ml for resistant for non-meningitis isolates.

*The percentage indicates the pneumococcal isolates resistant or intermediate among the 195 invasive pneumococcal isolates tested for antibiotic susceptibility

## Discussion

We demonstrate an extremely high, yet dynamic, burden of paediatric pneumococcal disease in rural Mozambique before PCV introduction. The overall incidence observed during the twelve year period examined was 245 cases of IPD per 100,000 CYR among children <5 years old, which is higher than what has been reported for this age group from other African sites. Consistent with studies from other locations [23–27], the greatest burden was observed among infants <12 months (479 per 100,000 CYR), followed by 12–23 month olds (390 per 100,000 CYR). Even these very high incidences, however, are likely an underestimate, since more than 50% of paediatric deaths in the study area occur at home with no previous hospital attendance [28]. With a case fatality proportion of 14% overall and 29% among meningitis cases, our findings highlight the importance of *S*. *pneumoniae* as a cause of death among young children and the need to prevent pneumococcal deaths in order to achieve Millennium Development Goal 4.

PCV10 was introduced in the routine infant immunization program in Mozambique in April, 2013. Extrapolating the observed incidence rates in 2012 to 4,332,000 Mozambican children <5 years of age in 2012 [14], we estimate 4,592 hospitalizations for IPD and 643 IPD in-hospital deaths among children <5 years olds nationwide occur each year. Assuming 65% of these IPD cases and 49% of the deaths could be prevented by PCV10, approximately 2,985 admissions and 315 deaths could be averted annually. Available data on PCV10 impact [29] and effectiveness [30] from other countries suggest that it is highly protective against vaccine-type IPD among young children; however, these studies have been conducted in places with relatively low HIV prevalence. Data on PCV effectiveness against IPD among HIV-infected children are limited and inconclusive [5, 31]. Continued surveillance for IPD in Mozambique, an area with a very high burden of both HIV and pneumococcal disease, will provide important data on the benefits of PCV10 introduction in populations with a high-HIV prevalence.

Based on the serotype results, PCV13 would offer protection against a higher proportion of IPD cases in Mozambique (83% versus 65% coverage for PCV10 and 78% coverage for PCV10+6A). Since the two serotypes that primarily account for the difference were 6A and 19A, the true difference between the coverage of the two formulations may depend upon cross-protection from vaccine serotypes (6B and 19F). PCV7, which included serotype 6B, was shown in a case-control study to be 76% (95% CI 39, 90) effective against IPD caused by serotype 6A [32] and rates of 6A IPD fell in variety of settings where PCV7 was introduced [33, 34]. Immunogenicity studies suggest that PCV10 should also provide cross-protection against serotype 6A [35, 36]. A case-control study in Brazil did not find significant PCV10 effectiveness against serotype 6A IPD, although power was limited [30]. That study did show an effectiveness of 82.2% (95% CI 10.7–96.4) against serotype 19A. However pneumococcal carriage data from Kenya show no impact of PCV10 introduction on carriage with serotype 6A or 19A [37]. The uncertainty about PCV10 cross protection underscores the need to monitor trends in non-vaccine serotypes following PCV10 introduction in Mozambique.

Our data also highlight the challenges in evaluating PCV10 impact due to the dynamic nature of pneumococcal disease. The incidence initially increased from 2001 to 2002, followed by a general downward trend over the course of the study period, albeit with year-to-year variation and relative increases observed in 2006 and 2011. The annual incidence ranged considerably from year to year, with a 5-fold difference between the lowest (106 per 100,000 CYR in 2012) and highest (531 per 100,000 CYR in 2002) annual rates measured. Certain pneumococcal serotypes (such as serotypes 1 and 5) can fluctuate over time, dramatically altering IPD trends. From 2003 on serotype 1 disease declined steadily, which may represent the resolution of a serotype 1 outbreak. The increase in overall IPD seen from 2001 to 2002 (before serotyping was routinely performed) is difficult to explain, but could also represent fluctuations in serotype 1. The general decline in IPD incidence over the course of the study period may have been influenced by a variety of factors including HIV prevalence and clinical management, socioeconomic and nutritional status. Improved access to antiretroviral treatment for HIV has been associated with a decline in IPD burden among HIV-infected children in South Africa [38] and HIV-infected adults in Malawi [39, 40]. Attempts to assess PCV10 impact by studying IPD trends over time must take into account pre-vaccine trends, and may be complemented by other methods (such as case-control vaccine effectiveness studies) or trends in other outcomes (i.e. pneumonia, pneumococcal carriage).

Antibiotic resistance data derived from this study are reassuring. Despite a high prevalence of penicillin resistance among pneumococcal isolates from other settings [41–43] we found less than 1% of strains to be resistant to beta-lactam antibiotics. These data support the empirical use of penicillin for management of pediatric pneumonia and suspected bacteremia/sepsis.

Several factors should be considered when interpreting these data. Only a single blood culture specimen was collected for each patient and lumbar punctures were performed for less than half of the children with suspect meningitis. Children with severe illness may die at home due to distance to health facilities and other barriers to care. HIV testing results were not available for the most IPD cases during this pre-PCV period and estimates of HIV prevalence in pediatric population are not available. The lack of data on HIV status make it impossible to calculate HIV-specific IPD incidence rates, and severely limits our understanding of driving forces behind the pre-PCV decline in IPD. Therefore these incidence estimates represent a minimum burden of disease. Nonetheless we report a remarkably high burden of invasive pneumococcal illness and death in this rural African setting with a high prevalence of HIV and limited access for antiretroviral treatment among children HIV-infected. Our findings suggest that recently introduced PCV10 will have a major impact on hospitalizations, death and health care costs in Mozambique. Efforts to measure that impact must take into account the complex nature of IPD and pre-vaccine trends in disease.

## Supporting information

S1 File (ZIP)
